# The expansion and validation of a new upper extremity item bank for the Patient-Reported Outcomes Measurement Information System® (PROMIS)

**DOI:** 10.1186/s41687-019-0158-6

**Published:** 2019-11-26

**Authors:** Aaron J. Kaat, Chester “ Trip” Buckenmaier, Karon F. Cook, Nan E. Rothrock, Benjamin D. Schalet, Richard C. Gershon, Mark S. Vrahas

**Affiliations:** 1 Department of Medical Social Sciences, Northwestern University, 625 N Michigan Ave Suite 2700, Chicago, IL 60611 USA; 2 Uniformed Services University, Defense & Veterans Center for Integrative Pain Management, 11300 Rockville Pike #709, Rockville, MD 20852 USA; 3 Department of Orthopaedics, Cedars-Sinai Medical Center, Mark Goodson Building, 444 S. San Vicente Blvd., #603, Los Angeles, CA 90048 USA

**Keywords:** Patient-reported outcomes measurement information system, PROMIS, Upper extremity, Physical function

## Abstract

**Background:**

The Patient-Reported Outcomes Measurement Information System® (PROMIS) includes a Physical Function (PF) item bank and an Upper Extremity (UE) item bank, which is composed of a subset of items from the PF bank. The UE item bank has few items and known ceiling effects. Therefore, this study aimed to expand the item bank to assess a wider range of functioning. With the additional content, other psychometric properties—improved content validity, item bank depth, range of measurement, and score reliability—were also evaluated. We convened an expert panel to review potential items, and then conducted psychometric analyses on both extant and newly-collected data.

**Results:**

Expert focus groups reviewed the PF item bank for items that were “sufficiently” related to upper extremity functioning for inclusion in the expanded UE item bank. The candidate item bank was quantitatively evaluated in a new sample of 600 people. The final items were calibrated in an aggregated dataset (*n* = 11,635) from two existing datasets, and the newly collected sample. The original UE item bank included 15 items. After expert review and quantitative evaluation, 31 items were added. The combined 46 items were calibrated using item response theory (IRT). Then computer adaptive tests (CATs) were simulated based off of the psychometric results. These indicated that the new UE item bank has an extended measurement range compared to the original version.

**Conclusions:**

The expanded PROMIS UE item bank assesses a wider range of upper extremity functioning compared to the initial UE item bank. However, ceiling effects remain a concern for unimpaired groups. The new UE item bank is recommended for individuals with known or suspected upper extremity limitations.

## Background

Physical function is one of the most important health outcomes when evaluating quality of life. It is a primary outcome for multiple musculoskeletal conditions [1], has been recommended as a target for drug development research in cancer [2], is strongly related to pain [3], and is a primary reason for disability applications [4]. However, debate persists as to the structure of physical function—specifically, whether it is a unitary construct or if it should be divided into subdomains. Various physical function measures differ on whether they are unidimensional [5] or multidimensional with various subscores [6–12]. In all of the multidimensional scales, measurement of upper extremity is distinct from other aspects of physical function such as mobility. Regardless, upper extremity and mobility are often tightly linked: being able to run errands, do chores, or lift heavy objects are likely diminished whether a person has either an upper extremity or a mobility concern. There has even been debate regarding whether upper extremity functioning should be further split into anatomical subdomains (e.g. hand, elbow, shoulder), though some have argued that there is little practical value in subdividing the measurement of upper extremity function by targeting narrower constructs [13, 14]. While a comprehensive review of the dimensionality of physical functioning is beyond the scope of this paper, recognition that upper extremity may be unique is of utmost importance, especially when evaluating clinical patients.

The Patient-Reported Outcomes Measurement Information System® (PROMIS) has contributed to this debate. PROMIS pediatric measures target physical function for children using two item banks: the PROMIS Pediatric Physical Function (PF)—Mobility and Pediatric PF—Upper Extremity (UE) [15]. Initial evidence for the unidimensionality of the adult PF measure was somewhat mixed, but was judged to be strong enough to warrant a single PROMIS-PF item bank [16, 17]. The adult PROMIS PF item bank was also found to be sufficiently unidimensional when translated, though the upper extremity items were less correlated with a convergent validity measure and the remainder of the PF item bank [18].

PROMIS has generally supported a single physical function score for adults. However, the PF item bank is composed of multiple facets [16–19], and as such, there is the threat of construct underrepresentation [20]. For this reason, the developers of the initial PROMIS-PF bank recommended the use of content balancing. Specifically, Rose and colleagues [16] suggest probing instrumental activities of daily living, upper extremity, back/neck, and lower extremity/mobility functioning, and cycling through these four subcategories of items to evaluate response consistency, reporting subcategory scores independently if consistency is low, though this particular recommendation has never been implemented. Clinicians and researchers have also requested separate upper and lower extremity function scores to match the facets relevant to their patient populations. To support this effort, PROMIS scientists used a strict definition to identify subsets of PF items exclusively measuring upper and lower extremity function (PROMIS UE, PROMIS Mobility) [19]. Small item subsets have different limitations—although they may better represent the content needed in specific clinical settings, they may be more prone to floor or ceiling effects.

A second concern regarding bifurcating the original PROMIS PF item bank into separate UE and Mobility item subsets is score comparability. Multiple forms and computer adaptive tests (CATs) from the same item bank should result in approximately equal scores for an individual. That is, if a person took more than one version, the scores should evidence a very high correlation, and any differences between the scores should be small, independent, and random (i.e. the scores should be *exchangeable*) [21–23]. Recent studies have evaluated the comparability for Mobility items and UE items in relation to overall PROMIS-PF. Scores were exchangeable in a sample of lower extremity trauma patients (correlation between Mobility-CAT and the 8-item short form [PFSF8a v1.0] was 0.91; mean difference approximately 1 T-score [24]). Scores were not exchangeable for other populations, including upper extremity trauma patients (correlations have ranged from 0.64 to 0.87 between the UE v1.0–v1.2 and PF scores [18, 19, 25]; with a mean score difference of approximately 8 T-score points favoring better outcomes on the PFSF8a than the UE-CAT for trauma patients [25]). These results suggest that the UE-CAT measured something different than the overall bank—at least among upper extremity orthopaedic trauma patients.

In the general population, physical function is likely unidimensional: either a person does well in all areas or a person struggles is all areas. In some clinical settings, physical function appears to be multi-dimensional. Upper extremity functioning is an additional dimension most relevant to those with known or suspected upper extremity limitations. Using a generic physical function measure alone among those with known or suspected upper extremity limitations may not be appropriate. For these reasons, this study aimed to improve the PROMIS UE item bank by: 1) improving the item bank depth and increase its content validity, which would also 2) increasing the range and precision of scores.

## Methods

### Item Bank development and qualitative review

There were 16 items in the UE v1.2 item bank [19]. An additional 61 items were identified from the PF v2.0 item bank as potentially related to upper extremity functioning [16, 26, 27]. These items were then rated by an expert panel composed of eight independent raters and two group facilitators with a broad range of expertise (e.g., orthopaedic surgeons, occupational therapists, rehabilitation psychologists, and measurement experts). The expert panel used to develop the UE v1.2 item bank [19] required items to reflect *pure* upper extremity functioning. The goal of the new expert panel was to ensure that the items were *sufficiently* related to upper extremity functioning. This criterion allowed inclusion of items that reflected both upper extremity and generic physical functioning provided that the experts viewed the items as clinically relevant to upper extremity patients. The experts independently rated all 61 candidate items prior to a consensus meeting during which differences were resolved.

### Data sources

Data were drawn from multiple sources. All candidate items were in the PROMIS PF v2.0 item bank [27] and therefore existing data from previous testing were available. New items were previously written for the PF v2.0 item bank that were intended to capture “elite” physical functioning skills—that is, skills above the ceiling of the v1.2 item bank, including elite upper extremity items—thus no new items were written for this project. The largest sample was drawn from the original PROMIS PF v1.0 dataset [16, 26], which included the centering sample, a second general population sample, and several clinical samples drawn from online and in-clinic samples. More information regarding the PF v1.0 dataset is described elsewhere [26]. The second source of data, the PF v2.0 sample, which was collected online utilizing the Op4G panel. It included four subsamples: a general population sample and three samples that represented expected poor, high, and very high physical function. Because very few individuals in the high and very high physical function groups had upper extremity limitations, data for these two groups were not used in the current analyses. However, the PF v1.0 and v2.0 datasets were limited insofar as most respondents completed between 3 and 7 UE items and no one had completed the entire candidate UE item bank. Therefore, the full candidate UE item bank was administered to a new online sample, collected through the Op4G panel, that included both individuals with self-reported functional limitations due to an upper extremity problem or concern, and those without such limitations. Table 1 provides demographics for the overall sample (*n* = 11,635). In total, there were 13 sub-samples across the three data sources. Demographic characteristics split by sub-sample are available in the Additional file 1.

**Table 1:** Sample demographics

Characteristic	Mean	SD
Age in Years	52.6	16.6
Characteristic	N	%
Gender		
Male	5410	46.5
Female	6224	53.5
Race^a^		
Caucasian	9931	85.4
African-American	1094	9.4
Asian-American	167	1.4
Native American or Alaskan Native	318	3.0
Native Hawaiian or Pacific Islander	27	0.3
Other	57	3.6
Ethnicity		
Non-Hispanic	10,572	91.1
Hispanic	1031	8.9
Education		
Less than HS	368	3.2
HS or GED	1909	16.4
Some College, Technical, or Vocational	4170	35.9
Bachelor’s Degree	2944	25.3
Advanced Degree	2238	19.2

^a^Note: Individuals were allowed to select more than one racial affiliation. The PF v2.0 online samples did not collect “Native American” or “Pacific Islander” and PF v1.0 did not collect “Other”

### Statistical analyses

Quantitative analyses with the UE v2.0 item bank followed standard PROMIS procedures [16, 28]. Insofar as existing data were limited by no participants having completed all items, unidimensionality, item fit, and local dependence only in the new online sample that responded to all items (*n* = 600). Though differential item functioning is recommended for scale development, as this had already been tested for the PROMIS PF v2.0 item bank it was not replicated here. Item fit was evaluated based on statistics from both factor analyses and IRT [16, 29, 30]. The analysis plan including removing items whose responses were not sufficiently unidimensional within the new item bank and removing one item of each item pair found to exhibit local dependence.

After finalizing items for the bank, a multiple group IRT calibration with the graded response model was conducted using flexMIRT [31, 32]. The centering sample from PF v1.0 set the reference scale, which was matched to the 2000 US census general population demographics [33]. Other groups were defined based on 1) clinical conditions for the PF v1.0 dataset, 2) physical functioning status for the PF v2.0 online samples, or 3) reporting or not reporting problems completing day-to-day tasks in the full bank dataset. For identification purposes, items were set as invariant across groups.

Finally, convergent validity between the UE v2.0 item bank was evaluated. Previous studies have found that PROMIS PF measures highly correlate with non-PROMIS measures (generally *r* > 0.80) [16, 18, 24, 25]. In the full bank dataset, the Flexilevel Scale of Shoulder Function Short Form (FLEX-SF) [34] and PROMIS PF v2.0 Short Form 8b (PFSF8b) were co-administered with the candidate UE item bank. We hypothesized that PROMIS UE scores would correlate well with the FLEX-SF (*r* > 0.80), as the scale is more closely related to upper extremity functioning, but that the scores would still correlate moderate-to-highly with the PFSF8b (*r* > 0.60), consistent with other evaluations of upper extremity compared to generic physical functioning [18, 19, 25].

### CAT simulation

After final item calibration, simulated CATs were obtained for the expanded UE item bank. Simulations were evenly drawn from one of five potential patient populations: a general population sample (mean = 50, SD = 10), a slightly impaired population (mean = 42, SD = 10; roughly similar to PF v1.0 Osteoarthritis), an impaired population with minimal variability (mean = 35, SD = 7; roughly similar to v2.0 Poor physical function), an impaired population with large variability (mean = 35, SD = 15), and a highly-impaired population (mean = 25, SD = 10; roughly similar to previously published “severe” fracture scores [25]). For each condition, 2500 responses were generated. This allowed examination of the average number of items needed for adequate measurement precision at various points along the upper extremity functioning continuum, item bank usage, and identification of optimal items for short form inclusion.

## Results

### Expert panel item review

The expert panel independently rated all 61 candidate items. Independent ratings were centrally aggregated and reviewed. Discrepancies were noted and resolved in a consensus meeting. All items addressed some aspect of upper extremity functioning, but the experts rated 28 items as insufficient for inclusion. Exclusions were made primarily for two reasons: Some referenced activities that required high levels of endurance (e.g. “Are you able to rake leaves or sweep for an hour without stopping to rest?”); others were judged to have too great a focus on mobility/lower extremity functioning (e.g. “Are you able to carry a suitcase up a flight of stairs?”).

### Statistical assumption checking

The remaining 33 candidate items were added to the existing 16-item UE item bank for quantitative evaluation (49 items total). The candidate item bank was broadly unidimensional (i.e., the scree plot, available by request from the first author, suggested one factor; the ratio of first-to-second eigenvalues was 34.6 to 3.7, and fit indices from a one-factor categorical confirmatory factor model, SRMR = 0.06, CFI = 0.99, RMSEA = 0.095). One item, however, had an extremely poor fit with the UE item bank and was removed (S-χ^2^ = 160.4, df = 81, *p* < .001). There was a locally-dependent item triplet (among the triplet, LD-χ^2^ > 34.0) that addressed lifting heavy objects. Two of these three items were eliminated. Following this, model fit significantly improved. No remaining items had a significant marginal χ^2^, and all of the positive LD-χ^2^ were reduced (most < 0.05, all < 0.15). The lingering items with elevated LD-χ^2^ were maintained as further removal of items would limit measurement of average and high-average upper extremity functioning.

### Multiple-group calibration and centering

Multiple-group calibration and centering was conducted on the remaining 46 items. Item parameters were centered on the same centering sample as the general PF item bank, thereby matching the 2000 US census. Additional file 1 provides the population distributions for each group on the T-score metric. With the item-level parameters, the average slope was 3.44 (SD = 0.72; range 1.81 to 4.95) and the average threshold/difficulty parameter was − 2.15 (SD = 0.73; range − 4.01 to − 0.06).

Consistent with expectations, individuals with limited upper extremity functioning scored below the population mean, as did other clinical groups with suspected poor upper extremity functioning. The two non-centering general population samples diverged: the PF v1.0 general population had a higher average upper extremity functioning (mean = 52) than the centering sample, but the PF v2.0 general population sample exhibited poorer average functioning (mean = 46) with significant variability in performance (SD = 12.3). Examination of alternative centering strategies (i.e., using all general population samples for centering without weighting to the PF v1.0 demographically-matched subsample) did not substantially alter these results.

### Convergent validity

The new full-sample data set allowed an evaluation of convergent validity between scores on a generic physical function measure (i.e. the PFSF8b) and a shoulder-specific one (the FLEX-SF). Table 2 shows the overall and group-specific correlations between the mean scores for these measures. The mean UE scores converged well with scores on both patient reported outcome measures. There were no statistically significant differences in the correlations by group with the PFSF8b; however, examining the difference between dependent correlations suggested that the correlation between UE and FLEX-SF was significantly lower in the Non-Limited group relative to the Upper Extremity Limited group (z = 2.87, *p <* .001) [35]. Nearly all individuals in the Upper Extremity Non-Limited group had mean scores near the maximum for the PROMIS measures and the FLEX-SF, whereas the average mean score for the Upper Extremity Limited group was consistently lower, suggesting a ceiling effect for those without impairments.

**Table 2:** Mean score correlations with the UE item bank

	Overall	UE Limited	UE Non-Limited
N	600	246	354
PFSF8b	0.79	0.72	0.69
FLEX-SF	0.70	0.69	0.55

*Abbreviations: PFSF8b* PROMIS Physical Function v2.0 Short Form 8b, *UE* Upper Extremity

### CAT simulation

CAT simulation information is provided in Table 3. The UE-CAT was able to measure a wide range of upper extremity functioning, with observed scores between 15 and 61, but it was best able to distinguish among individuals in the impaired ranges of functioning. This was expected given the average difficulty parameters for the items. However, ceiling effects occurred for a large proportion of the unimpaired simulees. If the CAT administration failed to reach the SE criterion of < 3.0 (on the T-score metric), it administered the maximum allowable number of items (12)—almost exclusively resulting in a final score of 61.

**Table 3:** Simulated CAT results

Simulation	N	Item Bank Utilization	Mean # items / respondent	Min T-Score [Min with SE < 3]	Max T-Score [Max with SE < 3]	Final SE > 3.0
M = 50, SD = 10 General Population	2500	41%	9.6	20.3 [20.3]	61.0 [49.1]	57%
M = 42, SD = 10 Slightly Impaired	2500	43%	7.4	14.6 [14.6]	61.0 [49.3]	29%
M = 35, SD = 7 Impaired, Minimal Variability	2500	43%	5.2	14.6 [14.6]	61.0 [49.2]	5%
M = 35, SD = 15 Impaired, Large Variability	2500	46%	6.3	14.6 [14.6]	61.0 [49.2]	20%
M = 25, SD = 10 Highly-Impaired	2500	46%	4.3	14.6 [14.6]	61.0 [48.0]	1%

*Abbreviations*: *M* Mean, *Max* Maximum, *Min* Minimum, *N* Number of simulated respondents, *SD* Standard deviation, *SE* Standard error

Each item was ranked by the percentage of simulated CATs in which it was administered and on its statistical properties to develop an upper extremity short form. Seven items were chosen that maximized the range of possible scores and minimized similar content. The PROMIS v2.0 Upper Extremity 7-item Short Form (UESF7a) with a sum score to T-Score conversion table is provided in the Additional file 1.

## Discussion

Considerable debate persists as to the dimensionality of physical function. The PROMIS PF item bank contains multiple facets of physical functioning, including items that target upper extremity content [16]. However, these items are not well-represented in existing short forms, nor are they frequently included in CAT administrations to respondents with higher function. The existing PROMIS PF item bank v2.0 provides excellent measurement properties for most individuals [16, 26, 27], but is less effective in measuring those with known or suspected upper extremity limitations.

This study extends the PROMIS UE item bank through expert consensus review and quantitative evaluations. Previous research has found that the overall PROMIS PF item bank provides higher estimates of functioning than the UE-CAT v1.2 [25], potentially due to construct underrepresentation for this population [20]. The new PROMIS v2.0 Upper Extremity item bank ensures upper extremity construct representation, thereby reducing the risk of biased score estimates, and converges well with other measures of upper extremity or whole-body physical functioning. The new item bank is centered on the same sample as the existing PROMIS PF v2.0 item bank. This allows comparisons for upper extremity functioning to those in the general population [33].

The main difference between the new UE v2.0 item bank and the overall PF v2.0 bank relates to item precision. PROMIS PF measures the broader physical function domain, to which these upper extremity items are statistically less informative. But when the items indicate upper extremity functioning only, they are statistically more informative. This is evident when comparing the slope parameters for the same items across the two banks. For example, PFA17, PFA18, and PFA20 have slope parameters of 2.43, 3.32, and 3.75, respectively, in the UE v2.0 item bank, whereas in the generic PF item bank, the slope parameters are reduced to 2.15, 2.47, and 2.70.

### Limitations

This study is not without limitations. Previous attempts to create upper and lower extremity subsets from the PROMIS PF item bank have found that there are few upper extremity items that required a high degree of ability and training (i.e. “elite” upper extremity functioning) [1]. When developing this UE item bank, three high ability tasks were eliminated due to misfit or local dependence. There is precedence in non-PROMIS measures to have separate subscales for basic and elite physical function tasks for non-upper extremity functioning [10], but this has not been widely adopted and was not necessary for the PROMIS PF v2.0 item bank [27]. Second, although the study aimed to increase the ceiling on the test, the absolute highest score has not changed substantially; rather, the new UE v2.0 item bank more effectively “fills-in” average ranges of functioning. The UE v1.2 item bank had an absolute highest score of 56, with the highest score meeting the SE stopping rule of 43; this has improved to 61 and 49, respectively. This means that 22% of the general population who did not have a satisfactorily reliable score can now be measured with adequate precision. There is a greater ability to evaluate low average to average upper extremity functioning than previously existed, but evaluation of high average and elite upper extremity functioning remains elusive. What is most likely is that elite skills are less generalizable across individuals. For example, endurance with throwing accuracy, multiple repetitions of push-ups or pull-ups, volleyball, sculpting, or even performing surgery could be considered elite upper extremity tasks, but ones with which few individuals have experience. This concern applies equally to other physical functioning subdomains, such as running marathons, endurance training, or cross-country skiing. More research, both qualitative and quantitative, is necessary to evaluate how well individuals can extrapolate to activities with which they have not had direct experience.

A second limitation relates to the CAT simulations. As was evident in Table 3, less than 50% of the items were chosen by the CAT using the administration rules PROMIS generally follows for adult banks. One of the rationales for this UE item bank extension was that the generic PF-CAT was not choosing upper extremity items, potentially leading to construct underrepresentation. One may argue that the unchosen items should be excluded: they are not chosen by the CAT and do not appear on the UESF7a. However, maintaining them in the item bank allows users to build a custom short form that would include those items—an option for all PROMIS domains.

A final limitation relates to the comparisons between previous versions of the UE item bank and this revision. Previous changes to both PROMIS PF and UE (e.g. from v1.0 to v1.1 or v1.2) involved removing items or minor rewording, such as adding metric units. The revision to UE v2.0 was more substantial, insofar as the items were recalibrated and moved to their own scoring metric. The new scoring metric remains correlated with the original PROMIS PF scoring metric (as evidenced by the high correlation between the generic PFSF8b short form and the UE v2.0 item bank). Future research should further evaluate whether the UE v2.0 has different responsiveness to treatment than the generic PF item bank, or if a direct comparison between the banks demonstrates other differences in validity.

### Future directions

An important next step in evaluating the UE item bank is assessment of its responsiveness over time. The PF item bank is often used clinically to measure changes following surgery (e.g. in orthopaedics), or declines over the course of a chronic disease (e.g. rheumatoid arthritis). We anticipate similar uses for the UE item bank, and therefore, it is important to assess its responsiveness.

Along with responsiveness evaluations, clinicians need to know how to interpret current scores and changes in scores from the UE item bank. While PROMIS offers some basic guidance regarding interpreting functional domains—namely classifying scores between 45 and 40 represent mild, between 30 and 40 as moderate, and below 30 as severe impairments—more guidance would be beneficial. For example, having normative information for clinical conditions would enhance score interpretation. Analogous efforts have been undertaken among cancer patients [36]. Meaningful score thresholds for PF and UE should also be considered for various patient populations.

## Conclusions

Improving health measurement requires ongoing and incremental changes to reflect best practices. While the overall PROMIS PF v2.0 item bank provides excellent measurement properties for most individuals, we recommend using the UE v2.0 item bank for individuals with known or suspected upper extremity limitations and among those where upper extremity concerns are the primary clinical emphasis. If an individual is no longer limited—that is, he or she is now on the ceiling of the UE item bank—and upper extremity problems are no longer the primary clinical concern, we recommend documenting the improved upper extremity functioning and switching to the generic PF v2.0 item bank for ongoing monitoring. The expanded PROMIS UE item bank v2.0 addresses clinical and research needs not previously met.

## Supplementary information

**Additional file 1: Table S1.** Demographic Characterisics by Group, **Table S2.** Population distributions on the UE Item Bank, and **Table S3.** PROMIS Upper Extremity v2.0 Short Form 7a.

## Data Availability

This study utilized portions of the PROMIS 1 Wave 1 dataset, which is available on the HealthMeasures Dataverse repository 10.7910/DVN/0NGAKG. New data collection on the Upper Extremity full bank administration is also on the HealthMeasures Dataverse repository 10.7910/DVN/IHNIRH.

## Abbreviations

CAT: Computer Adaptive Test
FLEX-SF: Flexilevel Scale of Shoulder Function Short Form
IRT: Item Response Theory
PF: PROMIS Physical Function Item Bank
PF-CAT: PROMIS Physical Function Computer Adaptive Test
PFSF8a: PROMIS Physical Function v1.0 Short Form 8a
PFSF8b: PROMIS Physical Function v2.0 Short Form 8b
PROMIS®: Patient-Reported Measurement Information System
UE: PROMIS Upper Extremity Item Bank
UE-CAT: PROMIS Upper Extremity Computer Adaptive Test
UESF7a: PROMIS Upper Extremity v2.0 Short Form 7a
