# Supplementary material for: The expansion and validation of a new upper extremity item bank for the Patient-Reported Outcomes Measurement Information System® (PROMIS)
Source: J Patient Rep Outcomes. 2019 Nov 26;3:69. doi: 10.1186/s41687-019-0158-6 (PMC6879697; doi:10.1186/s41687-019-0158-6)
Supplement: Supplementary file 1 — Additional file 1: Table S1. Demographic Characterisics by Group, Table S2. Population distributions on the UE Item Bank, and Table S3. PROMIS Upper Extremity v2.0 Short Form 7a. [file 41687_2019_158_MOESM1_ESM.pdf]

# Additional file 1: Table S1: Demographic Characteristics by Group

| Characteristic | PF v1.0 General Population (including Centering) | PF v1.0 Cancer | PF v1.0 Heart | PF v1.0 Osteo-Arthritis | PF v1.0 Rheumatoid Arthritis | PF v1.0 Psychiatric Disorders | PF v 1.0 Spinal Cord Injury | PF v1.0 Limited Health | PF v2.0 General Population | PF v2.0 Poor Physical Health | Full-Bank Limited Upper Extremity Functioning | Full-Bank Non-Limited Upper Exremity Functioning | Combined Sample |
|---|---|---|---|---|---|---|---|---|---|---|---|---|---|
| Number of Participants | 4,982 | 987 | 581 | 916 | 553 | 1,193 | 266 | 557 | 800 | 200 | 246 | 354 | 11,635 |
| Age -- Mean (SD) | 50.6 (18.3) | 62.2 (12.1) | 61.0 (11.8) | 61.4 (12.6) | 56.7 (11.0) | 48.2 (14.9) | 56.3 (11.9) | 48.3 (13.7) | 45.3 (16.6) | 52.4 (15.3) | 47.9 (13.7) | 50.2 (14.9) | 52.6 (16.6) |
| Gender -- Male -- N (%) | 2,124 (42.6%) | 633 (64.1%) | 397 (68.3%) | 330 (36.0%) | 264 (47.7%) | 582 (48.9%) | 201 (75.6%) | 207 (37.2%) | 392 (49.0%) | 70 (35.0%) | 76 (30.9%) | 134 (37.9%) | 5,410 (46.5%) |
| Gender -- Female -- N (%) | 2,858 (57.4%) | 354 (35.9%) | 184 (31.7%) | 586 (64.0%) | 289 (52.2%) | 610 (51.1%) | 65 (24.4%) | 350 (62.8%) | 408 (51.0%) | 130 (65.0%) | 170 (69.1%) | 220 (62.2%) | 6,224 (53.5%) |
| Race -- Caucasian -- N (%) | 4,020 (80.7%) | 927 (93.9%) | 531 (91.4%) | 851 (92.9%) | 502 (90.8%) | 998 (93.7%) | 253 (95.1%) | 530 (95.2%) | 621 (77.6%) | 172 (86.0%) | 218 (88.6%) | 308 (87.0%) | 9,931 (85.4%) |
| Race -- African-American -- N (%) | 638 (12.8%) | 37 (3.8%) | 22 (3.8%) | 40 (4.4%) | 22 (4.0%) | 144 (12.1%) | 6 (2.3%) | 12 (2.2%) | 106 (13.3%) | 21 (10.5%) | 15 (6.1%) | 31 (8.8%) | 1,094 (9.4%) |
| Race -- Asian-American -- N (%) | 48 (1.0%) | 5 (0.5%) | 9 (1.6%) | 4 (0.4%) | 5 (0.9%) | 10 (0.8%) | 3 (1.1%) | 5 (0.9%) | 47 (5.9%) | 3 (1.5%) | 10 (4.1%) | 18 (5.1%) | 167 (1.4%) |
| Race -- Native American or Alaskan Native -- N (%) | 168 (3.4%) | 21 (2.1%) | 14 (2.4%) | 26 (2.8%) | 12 (2.2%) | 33 (2.8%) | 16 (6.0%) | 19 (3.4%) | ** | ** | 4 (1.6%) | 5 (1.4%) | 318 (3.0%) |
| Race -- Native Hawaiian or Pacific Islander -- N (%) | 17 (0.3%) | 0 (0.0%) | 1 (0.2%) | 0 (0.0%) | 2 (0.4%) | 3 (0.3%) | 0 (0.0%) | 2 (0.4%) | ** | ** | 0 (0.0%) | 2 (0.6%) | 27 (0.3%) |
| Race -- Other -- N (%) | ** | ** | ** | ** | ** | ** | ** | ** | 38 (4.8%) | 6 (3.0%) | 4 (1.6%) | 9 (2.5%) | 57 (3.6%) |
| Ethnicity -- Hispanic -- N (%) | 4,317 (86.7%) | 957 (97.0%) | 561 (96.6%) | 885 (96.6%) | 519 (93.9%) | 1,100 (92.2%) | 258 (97.0%) | 541 (97.1%) | 712 (89.0%) | 184 (92.0%) | 214 (87.0%) | 324 (91.5%) | 10,572 (91.1%) |
| Ethnicity -- Non-Hispanic -- N (%) | 646 (13.0%) | 29 (2.9%) | 20 (3.4%) | 26 (2.8%) | 32 (5.8%) | 88 (7.4%) | 8 (3.0%) | 16 (2.9%) | 88 (11.0%) | 16 (8.0%) | 32 (13.0%) | 30 (8.5%) | 1,031 (8.9%) |
| Education -- Less than HS -- N (%) | 172 (3.5%) | 11 (1.1%) | 14 (2.4%) | 12 (1.3%) | 11 (2.0%) | 21 (1.8%) | 5 (1.9%) | 9 (1.6%) | 89 (11.1%) | 17 (8.5%) | 6 (2.4%) | 1 (0.3%) | 368 (3.2%) |
| Edu -- HS or GED -- N (%) | 1,033 (20.7%) | 54 (5.5%) | 64 (11.0%) | 83 (9.1%) | 52 (9.4%) | 145 (9.1%) | 31 (11.7%) | 56 (10.1%) | 231 (28.9%) | 47 (23.5%) | 53 (21.5%) | 60 (17.0%) | 1,909 (16.4%) |
| Edu -- Some College -- N (%) | 2,085 (41.9%) | 249 (25.2%) | 208 (35.8%) | 313 (34.2%) | 198 (35.8%) | 377 (31.6%) | 95 (35.7%) | 214 (38.4%) | 184 (23.0%) | 79 (39.5%) | 81 (32.9%) | 87 (24.6%) | 4,170 (35.9%) |
| Edu -- Bachelors -- N (%) | 1,006 (20.2%) | 325 (32.9%) | 159 (27.4%) | 244 (26.6%) | 161 (29.1%) | 356 (29.8%) | 70 (26.2%) | 160 (28.7%) | 208 (26.0%) | 35 (17.5%) | 80 (32.5%) | 140 (39.6%) | 2,944 (25.3%) |
| Edu -- Advanced Degree - N (%) | 681 (13.7%) | 348 (35.3%) | 136 (23.4%) | 264 (28.8%) | 131 (23.7%) | 293 (24.6%) | 65 (24.4%) | 118 (21.1%) | 88 (11.0%) | 22 (11.0%) | 26 (10.6%) | 66 (18.6%) | 2,238 (19.2%) |

** Not collected as part of this study

Additional file 1: Table S2: Population Distributions on the UE Item Bank

| **Group** | **Sample Size** | **Number of Items** | **T-Score Mean** | **T-Score SD** |
|---|---|---|---|---|
| PF v1.0 Centering Sample **(fixed)** | 2,172 | 43 | 50 | 10.0 |
| PF v1.0 General Population | 2,810 | 43 | 52 | 9.8 |
| PF v1.0 Cancer | 987 | 23 | 52 | 8.8 |
| PF v1.0 Heart | 581 | 4 | 46 | 10.1 |
| PF v1.0 Osteoarthritis | 916 | 14 | 42 | 6.0 |
| PF v1.0 Rheumatoid Arthritis | 553 | 17 | 45 | 8.7 |
| PF v1.0 Psychiatric Disorders | 1,193 | 14 | 47 | 7.0 |
| PF v1.0 Spinal Cord Injury | 266 | 5 | 40 | 6.5 |
| PF v1.0 Limited Health | 557 | 14 | 45 | 8.6 |
| PF v2.0 General Population | 800 | 19 | 46 | 12.3 |
| PF v2.0 Poor Physical Function | 200 | 19 | 36 | 6.0 |
| Full-Bank Limited Upper Extremity Functioning | 246 | 46 | 36 | 4.3 |
| Full-Bank Non-Limited Upper Extremity Functioning | 354 | 46 | 49 | 7.7 |

# Upper Extremity – Short Form 7a

**Please respond to each question or statement by marking one box per row.**

| | | Without any difficulty | With a little difficulty | With some difficulty | With much difficulty | Unable to do |
|---|---|---|---|---|---|---|
| PFA14r1 | Are you able to carry a heavy object (over 10 pounds /5 kg)? ..................................... | ☐ 5 | ☐ 4 | ☐ 3 | ☐ 2 | ☐ 1 |
| PFA34 | Are you able to wash your back? .............. | ☐ 5 | ☐ 4 | ☐ 3 | ☐ 2 | ☐ 1 |
| PFA36 | Are you able to put on and take off a coat or jacket? .................................................. | ☐ 5 | ☐ 4 | ☐ 3 | ☐ 2 | ☐ 1 |
| PFB13 | Are you able to carry a shopping bag or briefcase?.................................................. | ☐ 5 | ☐ 4 | ☐ 3 | ☐ 2 | ☐ 1 |
| PFB28r1 | Are you able to lift 10 pounds (5 kg) above your shoulder? ................................ | ☐ 5 | ☐ 4 | ☐ 3 | ☐ 2 | ☐ 1 |
| PFB34 | Are you able to change a light bulb overhead? ................................................. | ☐ 5 | ☐ 4 | ☐ 3 | ☐ 2 | ☐ 1 |
| PFM16 | Are you able to pass a 20-pound (10 kg) turkey or ham to other people at the table? ......................................................... | ☐ 5 | ☐ 4 | ☐ 3 | ☐ 2 | ☐ 1 |


© 2008-2016 PROMIS Health Organization and PROMIS Cooperative Group

# PROMIS Upper Extremity Short Form 7a
# Sum Score Conversion Table

All questions must be answered in order to produce a valid score using the scoring tables. If a participant has skipped a question, use the HealthMeasures Scoring Service (https://www.assessmentcenter.net/ac_scoringservice) to generate a final score.

| Raw Sum Score | T-Score | SD |
|---|---|---|
| 7 | 16.3 | 3.0 |
| 8 | 19.3 | 2.7 |
| 9 | 21.1 | 2.5 |
| 10 | 22.6 | 2.4 |
| 11 | 23.9 | 2.4 |
| 12 | 25.0 | 2.3 |
| 13 | 26.1 | 2.3 |
| 14 | 27.0 | 2.3 |
| 15 | 27.9 | 2.3 |
| 16 | 28.8 | 2.3 |
| 17 | 29.7 | 2.3 |
| 18 | 30.5 | 2.3 |
| 19 | 31.4 | 2.3 |
| 20 | 32.2 | 2.3 |
| 21 | 33.0 | 2.3 |
| 22 | 33.9 | 2.3 |
| 23 | 34.7 | 2.4 |
| 24 | 35.6 | 2.4 |
| 25 | 36.6 | 2.5 |
| 26 | 37.5 | 2.6 |
| 27 | 38.6 | 2.6 |
| 28 | 39.7 | 2.8 |
| 29 | 40.9 | 2.9 |
| 30 | 42.3 | 3.1 |
| 31 | 43.9 | 3.4 |
| 32 | 45.6 | 3.6 |
| 33 | 47.7 | 3.9 |
| 34 | 50.9 | 4.5 |
| 35 | 58.2 | 6.7 |
